# Therapeutic and prophylactic effect of intermittent preventive anti-malarial treatment in infants (IPTi) from Ghana and Gabon

**DOI:** 10.1186/1475-2875-7-198

**Published:** 2008-10-01

**Authors:** Jürgen May, Samuel Adjei, Wibke Busch, Julian J Gabor, Saadou Issifou, Robin Kobbe, Benno Kreuels, Bertrand Lell, Norbert G Schwarz, Ohene Adjei, Peter G Kremsner, Martin P Grobusch

**Affiliations:** 1 Research Group Infectious Disease Epidemiology, Bernhard Nocht Institute for Tropical Medicine, Bernhard-Nocht Straße 74, D-20359 Hamburg, Germany; 2 Kumasi Centre for Collaborative Research in Tropical Medicine, Kumasi, Ghana; 3 Ministry of Health/Ghana Health Service, District Health Directorate, Agona, Ashanti Region, Ghana; 4 Medical Research Unit, Albert Schweitzer Hospital, Lambaréné, Gabon; 5 Department of Parasitology, Institute of Tropical Medicine, University of Tübingen, Germany; 6 Clinical Research Group, Bernhard Nocht Institute for Tropical Medicine, Germany; 7 Infectious Diseases Unit, Division of Clinical Microbiology and Infectious Diseases, National Health Laboratory Services, Johannesburg, South Africa; 8 School of Pathology, Faculty of Health Sciences, University of the Witwatersrand, Johannesburg, South Africa

## Abstract

**Background:**

Intermittent preventive treatment in infants (IPTi) with sulphadoxine-pyrimethamine (SP) reduces the incidence of malaria episodes in young children. The exact mechanism by which the protective effect is mediated needs to be defined. This study aimed to investigate therapeutic, prophylactic, and possible exceeding effects of SP-based IPTi in two clinical trials.

**Methods:**

Protective efficacies from two IPTi trials performed in Kumasi, Ghana, and Lambaréné, Gabon, were assessed for overlapping time series of 61 days. For six-months periods after each of three IPTi doses a multivariate Poisson regression model with the respective cohort as co-variate was generated and effect modification of protective efficacy with time strata was evaluated by log-likelihood tests.

**Results:**

Protective efficacies were not significantly different between the two study cohorts. Study-cohort corrected protective efficacy was highest for the first 61 days after each IPTi application and decreased continuously. For the first 61 days after IPTi-1, IPTi-2, and IPTi-3 the protective efficacy was 71%, 44%, and 43%, respectively. A reduction of the malaria incidence rate was detectable for the first 60, 30 and 40 days after IPTi-1, IPTi-2 and IPTi-3 drug application, respectively. After IPTi-3 a higher risk for malaria could be seen after day 60. This effect was mainly based on the overwhelming influence of the Kumasi cohort.

**Conclusion:**

The results suggest that SP-based IPTi mainly works through a therapeutic and prophylactic effect over 30 to 60 days after drug application and that a sustained effect beyond post-treatment prophylaxis might be very low.

**Trial registration:**

Data analysis from clinical trials NCT ID # 00206739 (Kumasi Trial) and NCT ID # 00167843 (Lambaréné Trial), .

## Background

There is an urgent need to improve malaria control strategies for young African children who represent the population with highest risk for malaria-related morbidity and mortality [1]. In most parts of sub-Saharan Africa individual control measures are restricted to the reduction of transmission by the use of insecticide-treated bed nets [2] and the treatment of malaria attacks [3], often with an ineffective drug due to parasite drug resistance [4]. Promising new approaches such as multi-stage vaccines [5] and intermittent preventive treatment in infants (IPTi) [6] aim to inhibit the multiplication of *Plasmodium falciparum *after infection.

The concept of IPTi is to combine both therapeutic and prophylactic effects of an anti-malaria drug administered in intervals short enough to prevent the onset of disease and long enough to enable the development of protective immunity [7]. However, it is unclear which factors contribute to the effects of IPTi and whether an exceeding impact exists. It has been suggested that the optimal drug application schedule depends on the half-life of the drug used, the extent of parasitic resistances against the drug, and the incidence of malaria in the area [8].

The application scheme initially evaluated in Tanzania was pragmatically linked to the extended program of immunization (EPI) of the WHO at months 2, 3 and 9 and based on a weight-adjusted single dose of sulphadoxine-pyrimethamine (SP) [9]. The protective efficacy of the intervention to reduce malaria episodes was more than 50% in an area with a malaria incidence of 0.43 per person years. Subsequent studies in different African countries demonstrated a protective efficacy ranging between 17% and 25% [10-14].

Impairment of immune responses after routine vaccinations or clinical rebound effects known to occur after continuous chemoprophylaxis have not been reported [9,12,15,16], but concern exists about a possible rebound with higher incidence of malaria in two studies, particularily with high parasite densities [13,14]. The present study compared therapeutic, prophylactic, and exceeding effects of SP-based IPTi in study cohorts from Ghana and Gabon.

## Methods

This study was performed with study cohorts from two African sites with differing malaria endemicities. Main results of the clinical trials on protective efficacy and safety have been published elsewhere [10,11].

### Study areas

#### Kumasi cohort

The Afigya Sekyere district occupies an area of 714 km^2 ^in the forest belt of the Ashanti Region in Ghana close to the regional capital Kumasi. Its population was counted in the year 2000 census as 84,691 persons living in 16,173 households. Children under 12 months and under five years of age accounted for 4.0% and 18.6% of the population, respectively. The temperature varies from 20°C to 36°C with monthly rainfall varying from 2 mm in February to 400 mm in July. A major rainy season extends from April to August and a minor one from October to November. The local economy is based on cash crops such as cocoa, coffee and oil palm, although subsistence farming and small scale trading are the main sources of income.

Malaria is holoendemic in this area with an entomological inoculation rate (EIR) of about 400 infective bites per individual per year. The predominant *Plasmodium *species is *P. falciparum *(80–90%) and the principal malaria vectors are mosquitoes either of the *Anopheles gambiae *complex or *A. funestus*. During the time of the study the first-line drug for treatment of uncomplicated malaria in Ghana was changed from chloroquine to amodiaquine-artesunate. Chloroquine resistance of *P. falciparum *has spread nation-wide and the prevalence of molecular markers for sulphadoxine and pyrimethamine resistance is high in the study area [17]. The percentage of persons with at least one insecticide-treated bed net (ITN) in the Ashanti region is 1.6% [18].

#### Lambaréné cohort

Lambaréné is situated at the river Ogooué near the equator in the Moyen Ogooué province of Gabon, a typical Central African rain forest area. The average temperature is around 27°C and rainfall is high throughout the year with a minimum in July and August. The entomological inoculation rate is about 50 infectious bites per individual per year, mainly from vectors of the *A. gambiae *complex. Malaria transmission is perennial with limited seasonal fluctuations. Only the short rainy season produces an increase of parasite rates and densities.

The prevailing species is *P. falciparum*, responsible for more than 90% of all infections, together with some *P. malariae *and *P. ovale *infections. Parasite resistance against SP is intermediate and that against chloroquine is high and widespread [19,20]. Bed net use appeared to be high in the population and was 80% in the study group, but only a fraction of nets is impregnated and ITN coverage is estimated to be only 5% [11].

### Subjects and study design

#### Subjects

The data is based on randomized, placebo-controlled, double-blinded clinical trials performed in Ghana and Gabon. Detailed descriptions of the study design of both cohorts have been published elsewhere [10,11]. Briefly, monthly active follow-ups and passive visits between the age of three and 21 months were considered for this analysis. During every visit, the children were examined clinically by a staff physician and the medical history since the last visit was assessed. At treatment visits, scheduled at 3, 9 and 15 months of age, blood was drawn and the study drug was administered. A structured questionnaire and a case report form were completed and documented on each consultation. Bed net usage at the time of first study drug administration was assessed by interview and, whenever feasible, inspection of the house was performed.

Children received either a single dose of 250 mg sulphadoxine and 12.5 mg pyrimethamine or placebo (verum drug and placebo provided by Roche, Basel, Switzerland) at the age of 3, 9 and 15 months (IPTi-1, IPTi-2, IPTi-3, respectively). The substance was administrated orally by spoon after crushing the substance and mixing with water. Children were observed for 30 min, and a repeated dose was given if vomiting occurred. Treatment was stopped if the child vomited the second dose. Malaria attacks were treated with an oral combination of artesunate (4 mg/kg/day every 24 h over 3 days) and amodiaquine (10 mg/kg/day every 24 h over 3 days).

Inclusion criteria were (i) provision of parental written informed consent or witnessed oral consent in the case of illiteracy and (ii) permanent residency in the study area. Exclusion criteria were (i) known or suspected allergy to sulphonamides or pyrimethamine and/or signs and symptoms thereof and (ii) history of severe hepatic or renal dysfunction.

#### Kumasi cohort

Infants at the age of three months (n = 1070) were enrolled in the health posts of nine villages during their first contact for EPI vaccinations. Duration of the study was from January 2003 to September 2005 (NCT ID # NCT00206739, ). In the Kumasi cohort, a malaria episode was defined as the presence of any asexual *P. falciparum *parasitaemia (> 500 parasites/μl) and either a rectal temperature of at least 38.0°C or a history of fever during the last 48 h reported by the mother. Examination of blood films followed quality-controlled standardized procedures [10]. The protocol was approved by the Committee of Human Research, Publications and Ethics, School of Medical Sciences, Kwame Nkrumah University of Science and Technology, Kumasi, Ghana.

#### Lambaréné cohort

Newborns (n = 1189) were recruited on the maternity wards of the Albert Schweitzer Hospital (HAS) and the General Hospital (HG) in Lambaréné. Duration of the study was from 2002 to 2007 (NCT ID # 00167843, ). In the Lambaréné cohort, malaria episodes were defined as events of fever (rectal body temperature ≥ 38.5°C or fever reported to have occurred during the preceding 48 h) together with asexual *P. falciparum *parasitaemia. Parasitaemia was assessed according to the Lambaréné method, as described elsewhere [21]. The ethics committee of the International Foundation of the HAS approved the study.

### Statistical analysis

Data from questionnaires and forms were entered into a database software and cross-checked within 5 days of each visit at each site. All data of patients were treated confidentially. Copies of all data, and the original source documents (report forms) were retained at each study site.

**Figure 1:**
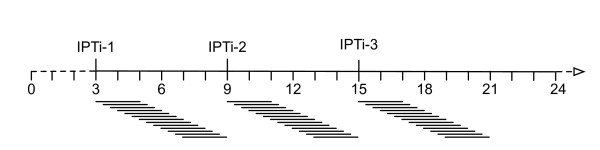
**Protective efficacies were calculated for overlapping series of 61-days periods for six months after each IPTi administration (IPTi-1, first IPTi dose at month 3; IPTi-2, second IPTi dose at month 9; IPTi-3, third IPTi dose at month 15). **The first 61-day period started at the time of an IPTi application, the last period ended at the time of the next IPTi application or 6 months after the last IPTi application (IPTi-3).

Analyses were performed with the STATA/MP software, version 10.0 (College Station, TX, USA). Protective efficacies were calculated for an overlapping series of 61-days periods (2-months periods) for intervals of six months after each SP administration (IPTi-1, IPTi-2, and IPTi-3, Figure 1). The first 61-day period started at the time of an IPTi application, the second 61-day period started at day 10 after the IPTi application, and so on. The last period ended at the time of the next IPTi application or six months after the last IPTi application (IPTi-3).

A treatment with a single dose of an anti-malarial drug without diagnosis will have a therapeutical effect only in individuals infected with drug-sensitive parasites. Accordingly, the potential therapeutic effect of IPTi was reflected by the proportion of children infected at time of drug administration not considering the drug sensitivity of the isolate. The true therapeutic effect can be considered as the potential therapeutic effect multiplied with the proportion of sensitive strains.

A single dose of an anti-malarial drug can only have a prophylactic effect if a reinfection with drug-sensitive parasites occurs during the time of effective drug levels. Accordingly, the potential prophylactic effect of IPTi was reflected by the proportion of children from the placebo arm (without prophylaxis) infected during the five weeks after drug administration without consideration of the drug sensitivity of the isolate.

Protective efficacy against multiple malaria episodes was determined by Poisson regression and defined as one minus rate ratio. For the analysis of the therapeutic effect, children were rated at risk for malaria during the complete time of follow-up. For the analysis of the prophylactic effect, children were not rated at risk for malaria for 21 days after preceding malaria episodes or after IPTi application. A multivariate Poisson regression model corrected for the study cohort (Kumasi or Lambaréné) was generated for the six months after each IPTi dose, and effect modification of protective efficacy with time strata was evaluated by log-likelihood tests. A significant protection was defined as a 95% confidence interval not including a 0% protective efficacy according to an alpha value p < 0.05.

## Results

### Basic data

Basic data at time of IPTi-1 show that the study cohorts and study arms were similar (Table 1).

**Table 1:** Basic data at first dose of IPTi

	Kumasi cohort		Lambaréné cohort	
	SP	Placebo	SP	Placebo

Participants, n	535	535	504	507
Age, mean days (sd)	90 (± 12)	89 (± 11)	96 (± 14)	97 (± 14)
Hb, mean g/dL (sd)	10.4 (± 1.4)^a^	10.3 (± 1.3)^b^	10.0 (± 1.2)^c^	9.8 (± 1.3)^d^
Anaemia^e^, %	2.1^a^	2.1^b^	0.9^c^	2.2^d^
Parasitaemia, geomean/μl^f^	681	779	790	1240

sd, standard deviation, SP, sulphadoxine-pyrimethamine

^a ^n = 531

^b ^n = 534

^c ^n = 461

^d ^n = 458

^e ^Hb < 7.5 g/dL

^f ^in those parasitaemic

#### Potential therapeutic effect of IPTi in dependence of application dose

In the Kumasi cohort, prevalences of parasitaemia at time of IPTi-1, IPTi-2, and IPTi-3, were 14.8%, 18.2%, and 24.3%, respectively, expressing the proportion of IPTi applications with a potential therapeutic effect on sensitive strains. Only 1.7% (18/1069) of children in the Kumasi cohort had malaria at the time of IPTi-1 application (SP or placebo) (Table 2). Out of these 18 children with malaria, eight were treated with SP and in 3 of these, malaria was detected during the 61 days after the drug application indicating either treatment failure or re-infection. At the time of the second and third IPTi application 8.2% and 9.1%, respectively, had malaria out of which 18.6% and 11.6% presented with treatment failure within 61 days. Only one malaria episode occurred during the first 21 days after an IPTi application (after IPTi-3 in Kumasi). In the Lambaréné cohort, prevalence of parasitaemia and malaria at the time of IPTi applications were always lower than 1.5% (Table 2).

**Table 2:** Probability of parasitaemia and malaria at time of IPTi application^a^

	IPTi-1		IPTi-2		IPTi-3	
	n	% (CI)	n	% (CI)	n	% (CI)

**Kumasi**	**n = 1069**		**n = 1012**		**n = 944**	
Parasitaemia	158	14.8 (12.7–17.1)	184	18.2 (15.9–20.7)	229	24.3 (21.6–27.1)
Asymptomatic	140	13.1 (11.1–15.3)	101	10.0 (8.2–12.0)	143	15.1 (12.9–17.6)
Symptomatic	18	1.7 (1.0–2.6)	83	8.2 (6.6–10.1)	86	9.1 (7.4–11.1)
						
**Lambaréné**	**n = 1011**		**n = 836**		**n = 703**	
Parasitaemia	6	0.6 (0.2–1.3)	9	1.1 (0.5–2.0)	10	1.4 (0.7–2.6)
Asymptomatic	4	0.4 (0.1–1.0)	3	0.4 (0.07–1.0)	0	0.0 (0.0–0.5)^b^
Symptomatic	2	0.2 (0.02–0.7)	6	0.7 (0.2–1.6)	10	1.4 (0.7–2.6)

CI, 95% confidence interval.

Denominator is the number of samples with available parasite assessment at time of IPTi application. The difference between the proportion of infected individuals in Kumasi and Lambaréné was always significant with at least p < 0.0006.

^a ^Application of SP or placebo at scheduled time of IPTi.

^b ^One-sided, 97.5% confidence interval.

#### Potential prophylactic effect after IPTi application

In children not being treated (placebo arm), the proportion of new malaria episodes during the 61 days after IPTi-1 was 5.4% (24/449) in the Kumasi cohort and 0.4% (2/484) in the Lambaréné cohort (Table 3). The incidence rate during this time in the Kumasi and Lambaréné cohort was 0.24 and 0.01 episodes per 61 days, respectively. In this analysis, the first 21 days after a malaria episode or malaria treatment were not counted as risk time for the assessment of new malaria episodes.

**Table 3:** Reinfection risk of untreated children during 61 days after scheduled time of IPTi application

Event^a^	IPTi-1		IPTi-2		IPTi-3	
	n	% (CI)	n	% (CI)	n	% (CI)

**Kumasi**	**n = 449**		**n = 428**		**n = 368**	
Parasitaemia	90	20.0 (16.4–24.1)	107	25.0 (21.0–29.4)	111	30.2 (25.5–35.1)
Asymptomatic	66	14.6 (11.6–18.3)	62	14.5 (11.3–18.2)	61	16.6 (12.9–20.8)
Symptomatic	24	5.4 (3.5–7.8)	45	10.5 (7.8–13.8)	50	13.6 (10.3–17.5)
						
**Lambaréné**	**n = 484**		**n = 412**		**n = 317**	
Parasitaemia	3	0.6 (0.1–1.8)	12	2.9 (1.5–5.0)	3	1.0 (0.2–2.7)
Asymptomatic	1	0.2 (0.0–1.1)	1	0.2 (0.0–1.3)^b^	0	0.0 (0.0–1.2)^b^
Symptomatic	2	0.4 (0.1–1.5)	11	2.7 (1.3–4.7)	3	1.0 (0.2–2.7)

CI, 95% confidence interval.

Denominator is the number of children from the placebo group without parasitaemia at scheduled time of IPTi application and with at least one available parasite assessment during the 61 days after IPTi application. The difference between the proportion of infected individuals in Kumasi and Lambaréné was always significant with at least p < 0.0001.

^a ^Nominators are number of events during 61 days after scheduled time of IPTi application.

^b ^One-sided, 97.5% confidence interval.

#### Protective efficacy in dependence of the time after IPTi application

**Figure 2:**
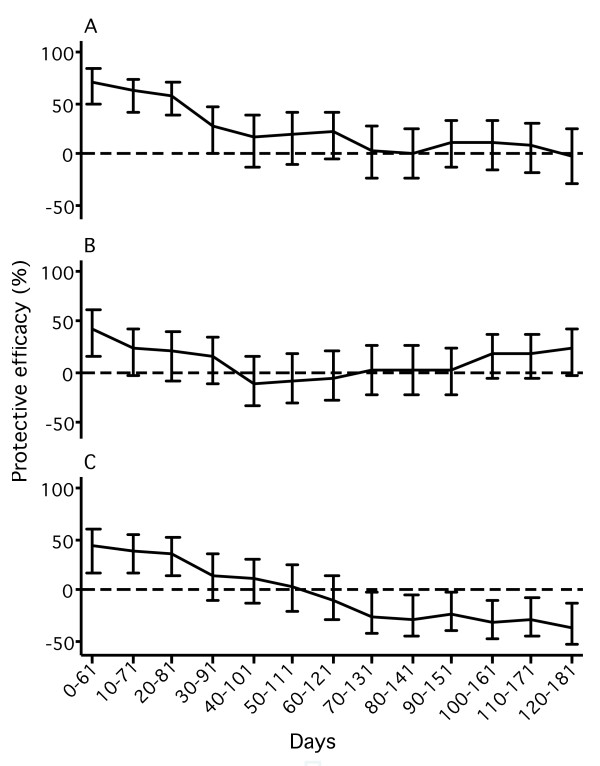
**Protective efficacies for intervals of 61 days for six months after each SP administration (A, IPTi-1; B, IPTi-2; C, IPTi-3).** Solid lines, protective efficacy; vertical bars, 95% confidence intervals. The first 61-day interval started at the time of an IPTi application and the last interval ended at the time of the next IPTi application or 6 months after the last IPTi application (IPTi-3). Protective efficacy against multiple malaria episodes determined by Poisson regression and defined as one minus rate ratio. Children were not rated at risk for malaria for 21 days after preceding malaria episodes or after antimalarial treatment.

Protective efficacies calculated for 61-days periods were not significantly different between the two study cohorts (tested with log likelihood tests) and were analysed in a pooled dataset with correction for the study cohort. Study-cohort corrected protective efficacy was highest for the first 61 days after each IPTi application and decreased continuously with time since drug application (first 61 days after IPTi-1, IPTi-2, and IPTi-3 the protective efficacy was 71%, 44%, and 43%, respectively; Figure 2). After IPTi-1 a significant protection was detectable until day 60 after drug administration. The effect disappeared after day 30 and 40 after IPTi-2 and IPTi-3, respectively. Sixty days after IPTi-3 an increased risk for malaria could be seen. This effect was mainly based on the overwhelming influence of the Kumasi cohort.

## Discussion

The efficacy of IPTi to protect from malaria episodes was between 17% and 62% in different African study areas resulting in an overall protective efficacy of about 25–30% [6,9-14]. The mechanism of how IPTi works is not fully understood and it has not been demonstrated whether protection is based exclusively on therapeutic and prophylactic or on additional exceeding effects [6]. The data collected at the two study sites with very different malaria incidences presented here shows a similar picture in terms of the protective effects. One limitation of the analysis is the very low number of malaria episodes in Lambaréné causing a reduced statistical power in this cohort.

First, the potential therapeutical efficacy, namely the probability of malaria at the time of IPTi application, is expected to be directly dependent on the malaria incidence in the area. Accordingly, it was about ten times higher in the Kumasi than in the Lambaréné cohort. The risk of malaria at time of SP application increased with age in both sites. From the data presented here one can derive that in highly endemic areas with relatively low frequencies of parasite resistance, the therapeutic efficacy may range from 1% to 25%.

Second, the potential prophylactic effect can be regarded as the probability for malaria during the time in which efficient blood levels are present. This probability is also directly dependent on the malaria incidence rate. Accordingly, it was significantly higher in Kumasi. In both study sites and in contrast to the therapeutic efficacy the theoretical prophylactic effect increased only slightly with age.

Third, and most importantly, the results presented here indicate a significant „post treatment prophylaxis" (PTP) against malaria episodes of IPTi, which was highest after IPTi-1 and lower after IPTi-2 and IPTi-3. Possible explanations for the decrease of the protective efficacy are a decrease of dosage per body weight with age, changes in the preexisting drug resistance, and the development of semi-immunity which may equalize the drug effects in both study arms [10].

The PTP decreased rapidly during the first weeks after IPTi administration and then disappeared. Sixty days after IPTi-3, the risk for malaria was increased in the Kumasi cohort and might indicate a rebound effect. Such a malaria rebound was not detected in the analyses performed according to the pre-defined analysis protocols of the clinical trials [8,11].

After IPTi-1, the dose with the highest protective efficacy, a prolonged protective effect was detectable 35 days after the complete elimination of the drugs, namely after five half-lives of sulfadoxine. Due to the synergy of the two components the effect of one treatment dose was estimated to last as long as 60 days against sensitive parasite strains [22,23]. In principle, the duration of the PTP might be compromised by the drug dose, pharmacokinetics of the drug used, and the resistance level of the parasites [22-24]. In one study from Northern Ghana the protective effect against malaria was 77% – 84% during the first month after each dose of IPTi and -11% – 43% during the second month, demonstrating a sharp decrease of the effect during the first weeks after drug application [13].

There was no evidence for a sustained protective effect of IPTi in the study cohorts analysed here. In the clinical trial from Tanzania such exceeding impact was inconsistently observed, depending on the analysis performed [9,25]. Only when children were included irrespective of malaria episodes in the first 10 months of life such an effect could be detected. Schellenberg *et al*. postulated that IPTi decreases the risk of a first malaria episode, which itself is a major risk factor for subsequent episodes [25]. An alternative explanation hinges on the assumption that *P. falciparum *and SP-based IPTi interact mainly in children with subtherapeutic drug levels when acquiring new infections. The authors hypothesized that the combined antiplasmodial effects of SP, fetal haemoglobin, and maternal immunity may prevent the development of new blood-stage infections into clinical attacks. Corresponding to the experimental observations of *P. yoelii *in mice, parasites could have further been attenuated *in vivo*, resulting in an enhanced opportunity to generate protective immune responses [26].

Intervention effects might be underestimated in both study cohorts because mild malaria episodes were treated early, which inherently comprises the risk of equalization of the study arms. This is of particular importance because amodiaquine, a long half-life drug which might exert similar effects as the study drug itself, was used in both cohorts for the treatment of uncomplicated malaria episodes [27,28]. Indeed, immune responses after single-dose SP applications in the Kumasi cohort indicated an underestimation of the protective efficacy of IPTi [29].

## Conclusion

The results suggest that the effect of SP-based IPTi is mainly therapeutic and prophylactic. It should be taken into account for the optimization of IPTi application schedules that sustained effects beyond prophylaxis cannot be expected in all settings and it remains unclear why such effects were found in certain studies but not in others. The optimal interval between two IPT applications in infants might be mainly determined by the probability of re-infections after the end of the synergistic drug effects, which is dependent on the prevalence of parasite drug resistance and the incidence of *P. falciparum *infections in a specific area [7,24]. The observation that the IPTi effect is mainly therapeutic and prophylactic might limit the selection of drugs eligible for IPTi to those that are long-acting. Though there are alternative candidate drugs that may be feasible [30], the long pharmacological half-lives of sulfadoxine and pyrimethamine render the combination suitable for IPTi [31]. Together with the unique property of being a one-dose anti-malaria drug, having a convenient safety profile, and being an inexpensive agent seems to favour SP for IPTi at least for the time being, provided that it is not used as the first line drug for treating malaria in the same area [30].

## Footnote

Recently, a secondary analysis of data from a cluster-randomized trial of SP-based IPTi in an area of intense seasonal transmission also demonstrated that protection against malaria lasts between 4 to 6 weeks, whereas the level of protection was higher during the first weeks [32]. The methodology of this analysis was very similar to that of the present work with the difference that the analysis period was not for the same time periods (61 days in the present analysis) but ended at time of the next IPTi application.

## Competing interests

The authors declare that they have no competing interests.

## Authors' contributions

All authors participated in design, implementation, field work, analysis and interpretation of the studies. Substantial input came from all investigators. For the Kumasi cohort, JM designed the study and was involved in all phases of the study and had full access to all the data in the study. JM takes responsibility for the integrity of the data and the accuracy of the data analysis. OA was the Principle Investigator. RK and SA were responsible for coordination of the field work. Further acquisition of data was performed by BK. Analysis of data of the manuscript was led by JM, RK, and WB. For the Lambaréné cohort, PGK, MPG, and BL designed the study. BL and MPG take responsibility for the integrity of the data and the accuracy of the data analysis. SI was the Principle Investigator. NGS and JJG were responsible for coordination of the field work. Writing of the manuscript was led by JM and MG, with all authors contributing significantly to the final version.
